# Effectiveness of supervised early exercise program in patients with arthroscopic rotator cuff repair: Study protocol clinical trial

**DOI:** 10.1097/MD.0000000000018846

**Published:** 2020-01-24

**Authors:** Héctor Gutiérrez-Espinoza, Felipe Araya-Quintanilla, Sebastian Pinto-Concha, Jonathan Zavala-González, Gonzalo Gana-Hervias, Iván Cavero-Redondo, Celia Álvarez-Bueno

**Affiliations:** a Rehabilitation and Health Research Center, CIRES, University of the Americas; b Physical Therapy Department, Clinical Hospital San Borja Arriaran; c Faculty of Health Sciences, University SEK; d Physical Therapy Department, Clinica Las Condes; e Adult Orthopedic Department, Clinical Hospital San Borja Arriaran, Santiago, Chile; f Universidad de Castilla-La Mancha, Health and Social Research Center, Cuenca, Spain; g Universidad Politécnica y Artística del Paraguay, Mayor Sebastián Bullo, Asunción, Paraguay.

**Keywords:** arthroscopy, electromyography, exercise therapy, randomized controlled trial, rotator cuff

## Abstract

**Background:**

Based on the available evidence, it is difficult to make a clinical decision about the best exercise program and to establish the most favorable time to start postoperative treatment after rotator cuff (RC) repair. The aim of this trial is to evaluate the effects of adding a supervised early exercise program to standard treatment for functional improvement and pain relief compared with standard treatment alone in patients with arthroscopic RC repair.

**Method/design:**

A total of 118 patients between the ages of 18 and 50 years with arthroscopic RC repair will be randomized to 2 treatment arms. The control group will receive a standard exercise program based on a consensus statement on shoulder rehabilitation developed by the American Society of Shoulder and Elbow Therapists. The intervention group will receive a supervised early exercise program in combination with standard treatment. This supervised exercise program will be based on electromyographic evidence. Three evaluations will be performed: before surgery, at 6 weeks, and at 12 weeks. The primary outcome measure will be the shoulder function by the Constant–Murley questionnaire, and the secondary outcome measures will be the upper limb function by the disabilities of the arm, shoulder, and hand questionnaire; pain by the visual analog scale; and the shoulder range of motion by a goniometer.

**Discussion:**

We hypothesize that patients who receive a supervised early exercise program in combination with standard treatment will benefit more in respect to shoulder function, pain reduction, and range of motion than those who receive a standard exercise program. If this is confirmed, our study can be used clinically to enhance the recovery of patients with arthroscopic RC repair.

**Trial registration:**

Brazilian registry of clinical trials UTN number U1111-1224-4143. Registered December 18, 2018.

## 1 Introduction

Rotator cuff (RC) disease is the most common etiology of shoulder pain, responsible for up to 70% of all shoulder-related visits to physicians.^[1,2]^ The prevalence of RC tears increases with age, from 9.7% in patients under 20 years to 62% in patients over 80 years. These rates are increased in symptomatic and after shoulder dislocation patients.^[3]^ Despite this widespread prevalence in the general population, optimal management remains controversial.^[4]^

Physical therapy is widely used for atraumatic tears, and several studies have demonstrated its reliable and success.^[5–10]^ However, physical therapy treatment does not result in the healing of the RC tear, and natural history studies have raised concerns about tear progression and irreversible fatty infiltration worsening over time.^[11–15]^ Surgery for RC disease is usually performed after conservative treatment has failed.^[16,17]^ Patients surgically treated return to work earlier and incur less cost burden when compared with patients treated nonoperatively.^[18]^ Additionally, successful outcomes following RC repair do not decrease at medium and long-term follow-up.^[19]^ Despite this, beneficial results have been reported for both conservative and surgical treatment of RC tears. A systematic review showed limited evidence that surgery is not more effective than conservative treatment in patients with RC tear.^[20]^ However, another systematic review showed statistically significant improvement in shoulder function and pain relief for patients managed surgically compared to those managed non-surgically, but these differences were small and did not meet the minimal difference considered clinically significant.^[21]^

Surgical options include partial repair and/or debridement, repair (open or arthroscopic), reconstruction (muscle transfer or processed tissue), and arthroplasty (hemi or reverse shoulder).^[22,23]^ The widespread use of arthroscopy has been related to a significant increase in RC repair procedures in recent decades,^[24]^ and currently arthroscopic repair has replaced open surgery and is now used to treat greater than 95% of all RC tears.^[25]^ Although the arthroscopic RC repair decreased pain, earlier functional recovery, and lower re-tears rates compared to open or mini-open procedures,^[26,27]^ a number of complications still occur in association with all types of RC repair.^[28]^ The overall rate for self-reported surgical complications after arthroscopic shoulder procedures is 7.9%,^[29]^ being the shoulder stiffness and residual pain the most reported.^[29,30]^

Following arthroscopic RC repair, a period of movement restriction is advised^[31]^; however, the optimal time of immobilization is unknown. It is common a practice to ask patients to use a sling for 6 weeks and avoid activities with the affected shoulder.^[32,33]^ This period is important to protect the tendon, allow healing, and prevent re-tear events.^[34]^ However, delayed motion may increase the risk of postoperative shoulder stiffness and muscle atrophy and delay functional recovery.^[32]^ Based on the available evidence, it is difficult to make a clinical decision about the best rehabilitation regime and establish the most favorable time to start the postoperative rehabilitation program.^[35]^ In recent years, there is still controversy regarding the influence of early versus delayed motion on stiffness and healing rate after RC repair.^[36]^ It is known that early motion rehabilitation increases range of motion (ROM) after RC repair,^[37–41]^ but the risk of re-tear is significantly higher compared to immobilization.^[38,39,42]^ Immobilization can result in stiffness of the shoulder, which can cause pain, functional limitations, and frustration for patients.^[43]^

Exercise is generally recommended to restore motion and shoulder function after arthroscopic RC repair.^[44]^ However, exercise prescription should be based on known muscle activity levels elicited during each respective exercise, as these are the best available estimates of stress placed on the RC tendon.^[45]^ Electromyography (EMG) via a percentage of maximal voluntary isometric contraction (MVIC) has been used as a pragmatic tool to guide postoperative rehabilitation progression by categorizing activation levels as low (0%–20% MVIC), moderate (21%–40% MVIC), high (41%– 60% MVIC), and very high (greater than 60% MVIC).^[44,46–48]^ From the perspective of rehabilitation, during the first 6 weeks after arthroscopic RC repair, early active exercises of the lower load (less than 20% of the MVIC) should be prescribed to improve function and motor performance of the shoulder without overloading the early surgical repair.^[44,45,47,48]^

This article reports the rationale and methods of a trial aimed to evaluate the effects of adding a supervised early exercise program to standard treatment on functional improvement and pain relief compared with standard treatment alone in patients with arthroscopic RC repair.

## 2 Method

### 2.1 Study design/setting

**Figure 1:**
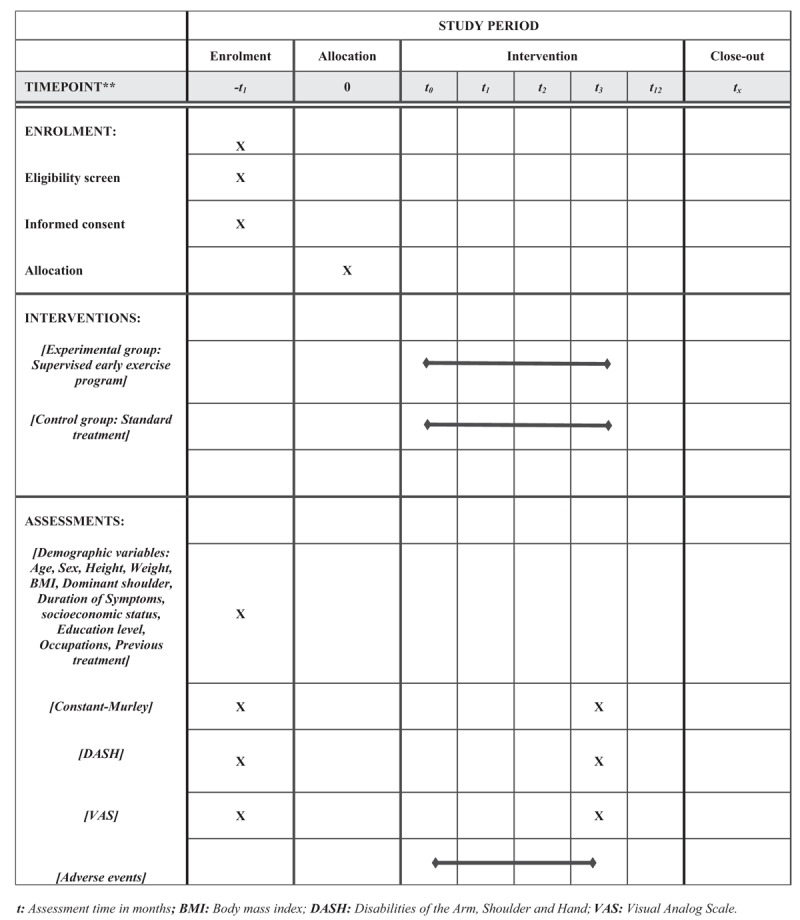
Standard protocol items: recommendations for interventional trials (SPIRIT) figure. BMI = body mass index, DASH = disabilities of the arm, shoulder, and hand, *t* = assessment time in months, VAS = visual analog scale.

This study will be a single-blinded, randomized controlled trial with 2 parallel groups. It will be conducted at the Physical Therapy Department of the Clinical Hospital San Borja Arriaran in Santiago, Chile. This protocol was written and based on Standard Protocol Items: Recommendations for Interventional Trials guidelines.^[49]^ The participants are residents who will be recruited mainly in the city of Santiago. The participants will be informed about the research, procedures, risks, and benefits by HGE (author of this protocol). If they agree, they will sign an informed consent form. Only those participants who read and agree to the protocol and who sign the informed consent form will take part of the study, following the schedule described in Figure 1.

### 2.2 Participants

A total of 118 patients with RC tear will be operated on by the Arthroscopic Surgery Team of the Clinical Hospital San Borja Arriaran. Before surgery, nonsurgical management failed (ie, persistent pain and/or disability following 3 months of conservative treatment, including analgesic or anti-inflammatory medications, intra-articular corticosteroids, activity modification, and physical therapy). Small to medium-sized (less than 3 cm) full-thickness supraspinatus tear, stage 2 or less fatty infiltration confirmed by preoperative ultrasound and magnetic resonance imaging, will be repaired arthroscopically with single-row suture. All patients will be treated with postoperative immobilization with sling and 500 mg of oral naproxen twice daily for 14 days; on the second day after surgery, they will be referred to the Physical Therapy Department.

#### 2.2.1 Inclusion criteria

To participate in the study, the subjects must meet the following inclusion criteria:

(1) older than 18 years old, and referred by the Adult Orthopedic Department with arthroscopic RC repair of a nonretracted isolated full-thickness supraspinatus tear,
(2) poor response to initial nonoperative treatment, and
(3) accept and sign the informed consent form.

#### 2.2.2 Exclusion criteria

Patients will be excluded if they meet the following criteria:

(1) large-sized RC tears (3–5 cm),
(2) massive^[50,51]^ or irreparable^[52]^ RC tears,
(3) anteroinferior labral (Bankart) or superior labrum anterior to posterior lesions,
(4) severe glenohumeral osteoarthritis,
(5) adhesive capsulitis, or
(6) previous surgery on the affected shoulder and re-tears of the RC.

### 2.3 Interventions

The control group will receive a standard exercise program based on the consensus statement on shoulder rehabilitation developed by the American Society of Shoulder and Elbow Therapists (ASSET).^[45]^ This exercise program proposes a 2-week period of strict immobilization and a staged introduction of protected, passive ROM during weeks 2 to 6 postoperative, followed by restoration of active ROM beginning at 6 weeks, with a gradual strengthening progression beginning at 12 weeks postoperative.^[45]^ In stage 1 (0–6 weeks), the first 2 weeks will be of strict immobilization with a sling, then a passive protected ROM with limits of <90° of forward elevation and <20° of external rotation will be performed during weeks 2 to 6. The specific components of this stage are the following:

(1) patient education: important points of emphasis in education include understanding of the pathology and procedure, time frame for recovery, the associated precautions during each stage of the treatment, and the importance of a home exercise program;
(2) cryotherapy: the subjects receiving cryotherapy in the first 10 days postoperatively^[53]^; and
(3) a home exercise program based on passive and self-assisted exercises.

In this first stage of rehabilitation, the exercises chosen for passive ROM should have levels of EMG muscle activity ≤15% and should be performed only in a gentle and comfortable manner. Four exercises will be performed: self-assisted supine forward elevation 60° to 90°^[54–56]^; self-assisted external rotation with stick at 20° of glenohumeral abduction^[54,55]^; active motions of the elbow, wrist, and hand (no external weight); and pendulum exercise.^[54,55,57]^ This will be performed once a day for 2 weeks.

In stage 2 (6–12 weeks), a supervised exercise program based on active or active-assistive ROM with limits of <120° of forward elevation and <30° of external rotation will be performed. The exercises within this category use gravity-minimized positions, such as supine or side lying and/or short lever arms to promote RC and deltoid balance.^[58,59]^ Six exercises will be performed: towel slide or horizontal dusting^[47,56]^; active-assistive ROM supine washcloth press-up^[56]^; active ROM supine press-up^[56]^; side-lying supported active elevation^[47]^; active ROM reclined wedge press-up^[56]^; and supine elastic band forward elevation.^[47]^

**Figure 2:**
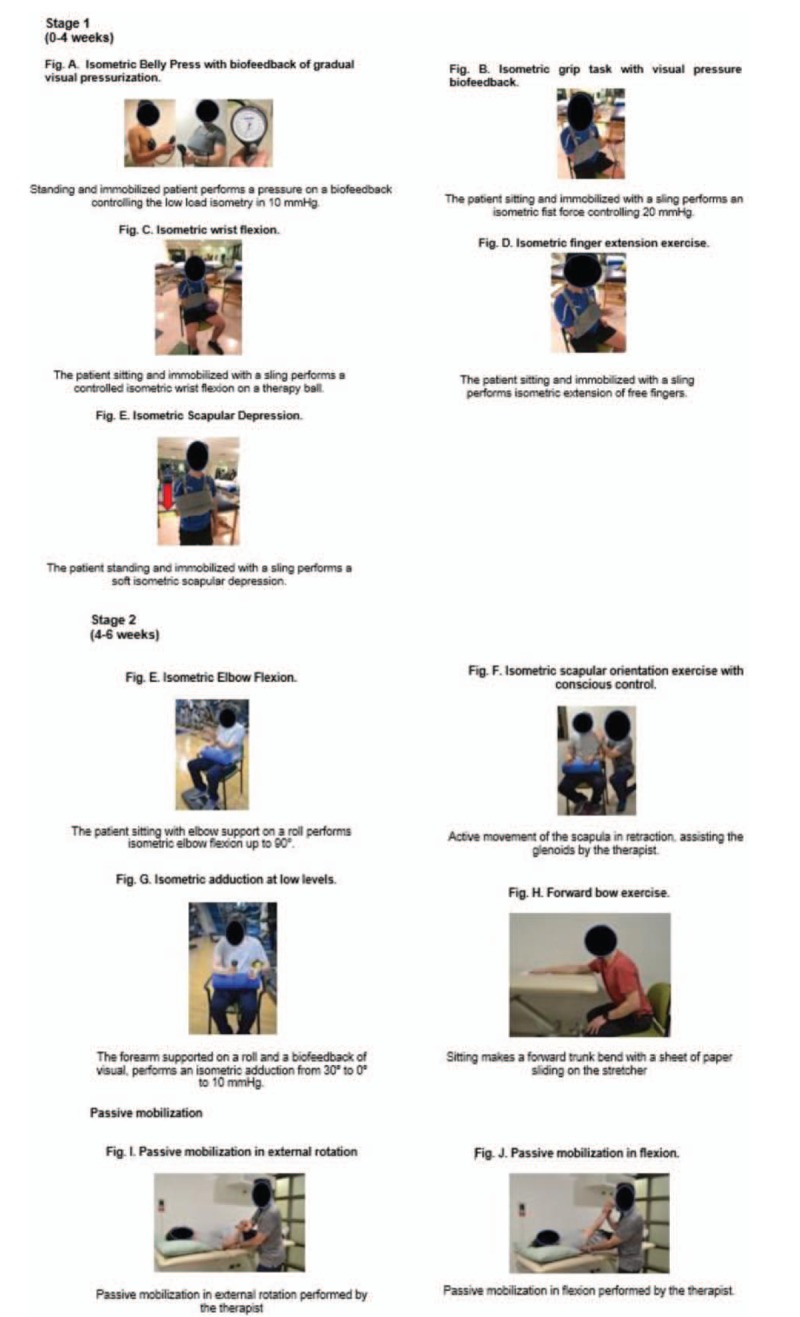
Detailed description of the supervised early exercise program.

The intervention group will receive a supervised early exercise program in combination with the standard exercise treatment. This exercise program will be based on EMG evidence (Fig. 2). Stage 1 (0–4 weeks), the first 2 weeks will be of strict immobilization with a sling, and 5 exercises will be performed generating remote or indirect activation of the RC with levels of EMG muscle activity ≤20% of MVIC: “belly press exercise with biofeedback” with an activation less than 20% MVIC for supraspinatus,^[60,61]^ “isometric hand grip task” with 90° elbow flexion with an activation less than 20% MVIC for supraspinatus,^[62,63]^ “isometric finger extension exercise” for infraspinatus muscle activation with 90° elbow flexion with significant activation of voluntary remote contraction,^[64]^ “isometric wrist flexion” to supraspinatus muscle activation in sitting position with an activation of less than 20% MVIC^[65]^, and “scapular depression” in sitting position with a 13% MVIC activation for supraspinatus.^[66]^

Stage 2 (4–6 weeks), 4 exercises will be performed: “isometric elbow flexion” for supraspinatus and upper subscapularis in sitting position,^[67]^ “scapular conscious control” for generating scapular retraction and scapular muscles activation,^[68]^ “isometric adduction” for infraspinatus and subscapularis activation,^[69]^ and “forward bow exercise” for supraspinatus and infraspinatus activation in sitting position.^[67]^ Additionally, 2 passive ROM exercises will be performed: passive external rotation and anterior flexion in supine position. These exercises will be assisted by at therapist.^[70]^

For control and intervention group the exercises should not produce pain; only mild to moderate pain levels (<4/10 on the visual analog scale [VAS]) are accepted after the session. Dose will be related to the goal of each exercise and will be adjusted in relation to the individual patient. From 8 to 10 repetitions of each exercise will be performed, with 5 to 10 seconds of task maintenance and 30 seconds to 1 minute of rest between each repetition. There will be 3 weekly sessions for 6 weeks.

### 2.4 Outcome measures

Baseline and postintervention outcome variables and potential confounders will be measured in both the intervention and control groups. Measurements will be performed before surgery, after 6 weeks, and the end of the program (12 weeks).

#### 2.4.1 Primary outcome measure

Shoulder function, as measured by the Constant–Murley questionnaire, will be the primary outcome.^[71,72]^ It consists of 4 sub-scales: 2 based on an interview with the patient regarding pain (a maximum of 15 points will be assigned) and activities of daily living (20 points), and the other 2 based on a physical examination about active ROM (40 points) and muscular strength (25 points), up to a maximum score of 100 points.^[71]^ This questionnaire shows a high correlation with other scales and shoulder-specific questionnaires, also shows high reliability and sensitivity to detect postintervention changes in a wide variety of shoulder pathologies.^[73]^

#### 2.4.2 Secondary outcome measures

Upper limb function, as measured by the disabilities of the arm, shoulder, and hand questionnaire, will be the secondary outcome.^[74]^ This is a self-administered questionnaire with 30 items related to

(1) the degree of difficulty during the previous week when performing various physical activities due to problems in the shoulder, arm, or hand (21 items);
(2) the severity of each of the symptoms, activity-related pain, tingling, weakness, and stiffness (5 items); and
(3) the effect of the problem on social activities, work, sleep, and psychological impact (4 items).

Each item could range from 1 (without difficulty to perform, without symptoms, or without impact) to 5 (unable to do, very severe, or high-impact symptom); and the final score could range from 0 to 100 (the higher the score, the greater the disability). A transcultural adaptation to the Spanish language has been made, whose version showed excellent results regarding validity, reliability, and sensitivity to change.^[75]^

Pain intensity at rest and movement will be assessed using the VAS, which consists of a horizontal line 10 cm in length, where the left end represents 0 or “painless” and the right end 10 or “worst pain imaginable.” The patient will be asked to mark with a vertical line the magnitude of the pain that feels at the time of the evaluation. It is a 1-dimensional, simple, and reproducible assessment method.^[76]^

A universal goniometer (precision: ±2°) will be used to assess the passive ROM of the shoulder in external rotation, abduction, scaption (elevation in the scapular plane), and flexion. External rotation will be measured with the patient in supine position, with the arm at the side of the trunk, the elbow flexed at 90°, and the forearm at neutral pronation/supination. The goniometer axis will be placed on the olecranon, the fixed arm perpendicular to the ground, and the movable arm aligned with the ulna, using the styloid apophysis of the ulna as a reference. The patient will be asked to external rotate the shoulder. Abduction will be measured with the patient in supine position, the shoulder in external rotation with the palm of the hand facing up, and the elbow in extension. The goniometer axis will be placed on the acromion, with the fixed arm parallel to the midline of the sternum and the movable arm aligned with the middle line of the humerus, using the epicondyle as a reference. The patient will abduct the arm until the onset of pain.

Scaption (elevation in the scapular plane) will be measured with the patient sitting in a chair with the trunk supported. The goniometer axis will be placed in the glenohumeral joint with the fixed arm perpendicular to the ground and the movable arm aligned with the longitudinal midline of the humerus, using the lateral epicondyle as a reference. The researcher will ask the patient to actively move the arm in the scapular plane through a small arc of movement, limited by a platform that defines the plane of movement.^[77]^ Flexion will be measured with the patient in supine position; shoulder at 0° of abduction, adduction, and rotation; elbow in extension; forearm in neutral pronation/supination; and with the palm of the hand towards the body. The goniometer axis will be placed in the glenohumeral joint with the fixed arm aligned with the middle line of the trunk and the movable arm aligned with the longitudinal midline of the humerus, using the lateral epicondyle as a reference. The patient will be asked to flex the shoulder and stop exactly at the onset of pain.^[78]^ Each movement will be measured 3 times, and the average of these measurements will be used for the analysis. Goniometer has good intra-rater reliability (intraclass correlation coefficient 0.91–0.99) when consistent body landmarks were used.^[79]^

#### 2.4.3 Potential confounders

Comorbidities: comorbidities that affect RC healing and postoperative outcomes such as hypercholesterolemia,^[16,80]^ diabetes,^[81–83]^ and smoking^[84,85]^ will be registered.

Clinical variables: the affected dominant shoulder, duration of symptoms (months), and previous treatments received in the last 3 months (supervised physical therapy, exercises only, use of paracetamol/nonsteroidal anti-inflammatory drugs, and use of opioids) will be registered.

Anthropometry and body composition: Weight will be measured with the patient barefoot and in light clothing. Height will be measured using a wall stadiometer, with the patient barefoot and upright and with the sagittal midline touching the back board. Body mass index will be calculated as weight in kg divided by the square of the height in meters.

Socio-economic status: Education level will be classified as primary education (functionally illiterate, without any studies, or those who have not completed primary education), middle education (primary education, high school/secondary, or baccalaureate education), and university education (college or PhD degree). Occupation will be categorized as heavy load, light load, and sick leave in the last month.

Daily movement behavior (DMB): defined as a construct including; physical activity, sedentary behaviors and sleep time. DMB will be measured by using the Xiaomi MI Band 3 Smart Bracelet, which provides information on physical activity (steps per day), sleep time, and sedentary time.

### 2.5 Sample size calculation

The sample size for this trial is based on an expected mean difference between groups of 11 points of the Constant–Murley questionnaire, which is the minimum clinically important difference.^[86]^ The mean assumed for the calculation was 63.3 with a standard deviation of 15 points based on results of other randomized clinical trials.^[87]^ To detect this difference between both treatments, with a value of *α* = 0.05 (probability of committing a type I error) and a statistical power of 95%, a minimum of 49 patients per group is needed. This minimal sample size estimate has been increased by 20% after considering the potential dropouts, finally including 58 patients for each group. Accordingly, the proposed experimental hypothesis is that there will be a difference of at least 11 points in the Constant–Murley questionnaire in the intervention group versus the control group. The sample size was determined using the Stata SE software, version 15 (StataCorp, College Station, TX).

### 2.6 Recruitment

Recruitment of the participants began in September 2019 and is expected to finish in March 2021. Before inviting participants to sign the informed consent form, information on the study goals and procedures will be provided verbally. The participants will also be invited to raise questions or doubts on any aspect of the study. Data confidentiality guarantees will be provided to participants by the principal investigator. Written consent will be obtained from all participants before registration, and participants may withdraw from the trial at any point in time without penalties. The written consent form includes information regarding the background and purpose of the study, therapeutic interventions, outcomes, and the expected benefits and drawbacks.

### 2.7 Randomization and blinding

**Figure 3:**
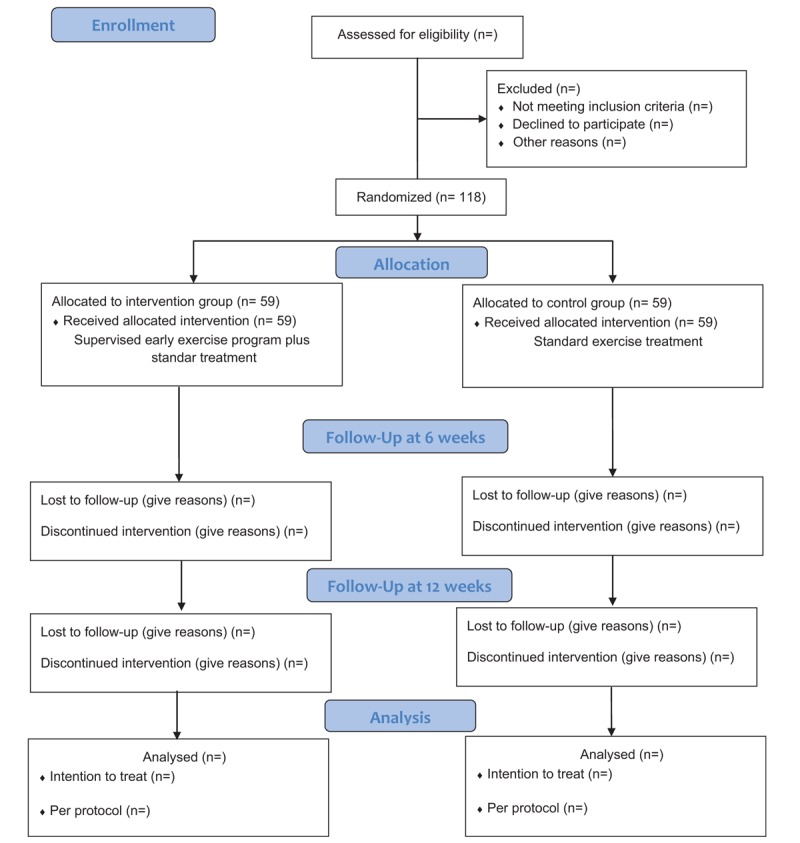
Flow diagram of patients through phases of clinical study.

Participants will be randomly allocated to the 2 groups through a sequence of numbers generated by a computer program before starting the selection process. The group assigned to each patient will be kept in a sealed envelope with the objective of concealing the assignment to the researcher, who will decide on the entry of subjects to the study (Fig. 3). Given the nature of the interventions, the physiotherapists, and the patients, blinding will not be possible. However, the evaluator and statistician will be blinded to which group the subjects evaluated will belong.

### 2.8 Data management

Information obtained from the evaluation of each participant will be recorded on a paper print-out. The information will then be handwritten on a paper document case report form and entered into an Excel file for future statistical analyses. In accordance with the Personal Information Protection Act, the names of all participants will not be disclosed, and a unique identifier number given during the trial will be used to identify participants. All of the participants will be informed that the clinical data obtained in the trial will be stored in a computer and will be handled with confidentiality. The participants’ written consent will be stored by the principal investigator.

### 2.9 Statistical analysis

The continuous variables will be presented as means and standard deviations, and categorical variables as number and percentage. To determine whether parametric statistical tests are appropriate for use in data analysis, the fitting to normal distribution will be evaluated using both statistical (Shapiro–Wilk test) and graphical (normal probability plot) methods. To examine baseline differences of the 2 groups, a 1-way analysis of variance (for continuous variables) and Chi-square tests (for categorical variables) will be conducted. Repeated measures analysis of covariance will be performed on outcome variables, with point of measurement (3 levels: preintervention, 6 weeks postintervention, and 12 weeks postintervention) as the within-subject factor and with type of intervention (2 levels: supervised early exercise program in combination with standard treatment versus standard treatment alone). The covariates were baseline values of each outcome variable.

Data will be processed independently by 2 researchers, and inconsistences will be detected using the VALIDATE command of Epi Info (CDC) software. After checking the truthfulness of outliers and extreme values, these will be winsorized using below the 1st percentile and above the 99th percentile of the distribution of variables. Before conducting the study, and after considering the nature of the missing data in patients with incomplete entries, these will be imputed using chained equations.

### 2.10 Harms

To collect, assess, report, and manage the potential adverse effects of the interventions, at the beginning and end of each treatment session, patients from both groups will have a logbook available. According to the informed consent for patients, those who show an increase in symptoms within 48 hours after the session will require an immediate evaluation by an orthopedic surgeon.

### 2.11 Ethics

The study will be conducted under the Declaration of Helsinki principles,^[88]^ as well as following the norms of good clinical practice. The study protocol has been approved by the Ethical Committee of the Central Metropolitan Health Service of Chile. This research was registered in the Brazilian Registry of Clinical Trials with the number U1111-1224-4143.

## 3 Discussion

The aim of this article is to describe the rationale and methods of a randomized controlled trial to test the effectiveness of 2 exercise programs in adult with arthroscopic RC repair. The control group will receive a standard exercise program based on a consensus statement on shoulder rehabilitation developed by the ASSET.^[45]^ The intervention group will receive a supervised early exercise program in combination with standard treatment. This supervised exercise program will be based on EMG evidence.

Despite the high incidence of surgery for RC repair, there is no consensus on the most effective approach for postoperative rehabilitation^[89,90]^ due to available evidence on timing and the load on the recovery process of tendon healing.^[34]^ Studies have shown that early motion increases ROM after RC repair^[37–41]^; however, the risk of re-tear is significantly higher compared with immobilization.^[38,39,42]^ Additionally, immobilization can result in shoulder stiffness, pain, functional limitations, and frustration for patients.^[43]^ Therefore, it is difficult to take a clinical decision about the best rehabilitation program. Nevertheless, the influence of these protocols on tendon healing and functional outcome is not well known.^[91–93]^

Usually, orthopedic surgeons prescribe a short period of immobilization and early passive mobilization to minimize stiffness; however, the type of immobilization, position, and shoulder motion remain unclear for tendon healing.^[42,94,95]^ Second, a home exercise program has not been developed, because factors such as age, tobacco use, comorbidities, and intrinsic tendon characteristics can be confounding factors for prescribe a home exercise program.^[40,42,95]^

Currently, strict immobilization time ranges from 0 to 6 weeks, where exercises are not prescribed to protect the surgery.^[90]^ Evidence needs from protocols describing important characteristics of a supervised early exercise program such as; an indirect and low load neuromuscular activation of the RC muscles, according to previous EMG studies, the load is less than 20% MVIC without overloading the repaired tendon.^[44,45,47,48]^ This protocol also describes other characteristic, a short duration isometric contraction muscular without compressive load in tendon, with a hypoalgesic action.^[96]^

Furthermore, this isometric exercise has other characteristics. First, this neuromuscular activity does not cause damage to the repaired tendon. Second, the short duration and low load do not have the possibility of generating muscle fatigue. And third, the exercises are supervised with a biofeedback based on motor skills training to reorganize cortical plasticity and achieve motor learning.^[97–99]^

To strengthen the reliability of the results, important methodological factors have been considered when planning this study. To avoid selection bias, a clinical and imaging diagnosis of RC will be considered, including echotomography and magnetic resonance imaging; and participants will randomly be assigned to the groups through a hidden allocation sequence. Furthermore, adjusting the sample size for possible losses or dropouts and increasing the number of patients recruited by 20% were considered; in the event of loss or withdrawals, statistical analysis will be carried out by protocol and intention to treat. To minimize measurement bias, all evaluations will be performed by 2 physiotherapists outside the research team, who will also remain blinded in relation to the treatment groups; the statistician will remain blinded to the group assignment of the participants. Finally, the outcome measures are suitable and frequently used in clinical practice, and they have good levels of validity and reliability.

Our study has some limitations. One important limitation is that the supervised early exercise program will be based on EMG evidence and has not yet been validated. Furthermore, the absence of follow-up once both treatments will be finalized, which does not allow establishment of the effectiveness of the therapeutic intervention in the long term. Finally, blinding of physiotherapists and patients was not achievable given the nature of the interventions studied.

To the best of our knowledge, this is the first clinical trial that studies a supervised early exercise program based on EMG evidence in combination with the standard exercise program based on the ASSET consensus statement, compared to a standard exercise program alone. The results of this study will add evidence to the limited and controversial body of knowledge related to the effectiveness of the different modalities of therapeutic exercises that are prescribed for patients with arthroscopic RC repair.

## Acknowledgment

The investigators would like to thank Mrs. Hernan Cañon Jones for her administrative support at our investigation.

## Author contributions

**Conceptualization:** Héctor Gutiérrez-Espinoza, Felipe Araya-Quintanilla, Sebastian Pinto-Concha, Celia Álvarez-Bueno, Iván Cavero-Redondo.

**Data curation:** Celia Álvarez-Bueno.

**Formal analysis:** Gonzalo Gana-Hervias, Celia Álvarez-Bueno, Iván Cavero-Redondo.

**Investigation:** Héctor Gutiérrez-Espinoza, Jonathan Zavala-González, Gonzalo Gana-Hervias, Sebastian Pinto-Concha.

**Methodology:** Héctor Gutiérrez-Espinoza, Felipe Araya-Quintanilla, Jonathan Zavala-González.

**Project administration:** Héctor Gutiérrez-Espinoza, Gonzalo Gana-Hervias, Celia Álvarez-Bueno.

**Supervision:** Felipe Araya-Quintanilla, Jonathan Zavala-González, Gonzalo Gana-Hervias, Iván Cavero-Redondo.

**Validation:** Iván Cavero-Redondo.

**Visualization:** Jonathan Zavala-González.

**Writing – original draft:** Héctor Gutiérrez-Espinoza, Felipe Araya-Quintanilla, Sebastian Pinto-Concha, Celia Álvarez- Bueno, Iván Cavero-Redondo.

Iván Cavero-Redondo orcid: 0000-0003-2617-0430.
